# Synthesis and Biological Evaluation of Novel 2-Arylalkylthio-5-iodine-6-substituted-benzyl-pyrimidine-4(3*H*)-ones as Potent HIV-1 Non-Nucleoside Reverse Transcriptase Inhibitors

**DOI:** 10.3390/molecules19067104

**Published:** 2014-05-30

**Authors:** Liang Zhang, Xiaowan Tang, Yuanyuan Cao, Shaotong Wu, Yu Zhang, Jianxiong Zhao, Ying Guo, Chao Tian, Zhili Zhang, Junyi Liu, Xiaowei Wang

**Affiliations:** 1 Department of Chemical Biology, School of Pharmaceutical Sciences, Peking University, Beijing 100191, China; 2 State Key Laboratory of Natural and Biomimetic Drugs, Peking University, Beijing 100191, China

**Keywords:** HIV, NNRTIs, S-DABOs, IC_50_, docking

## Abstract

A novel series of 2-arylalkylthio-5-iodine-6-substitutedbenzyl-pyrimidine-4(3*H*)-ones (S-DABOs) **8a**–**x** had been synthesized via an efficient method. Their biological activity against HIV virus and RT assay were evaluated. Some compounds, especially **8h**, **8l** and **8n**, displayed promising activity against HIV-1 RT with IC_50_ values in a range of 0.41 μM to 0.71 μM, which were much better than that of nevirapine. Molecular modeling studies revealed that the binding mode would be affected via forming an additional hydrogen bond by incorporating an oxygen atom on the C-2 side chain. The biological activity was in accordance with the docking results.

## 1. Introduction

**Figure 1:**
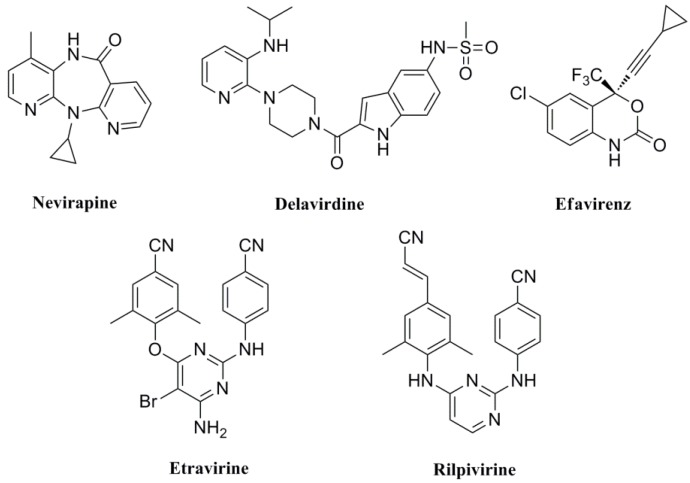
Structures of NNRTI drugs.

The reverse transcriptase (RT) of HIV-1, being an essential enzyme in the replication of the virus, is recognized as a great potential target for designing antiretroviral drugs [1]. In recent years, enormous efforts have been made to develop RT inhibitors. In particular, the non-nucleoside reverse transcriptase inhibitors (NNRTIs), a class of components of HAART, have received special attention due to their unique antiviral potency with generally low toxicity and favorable pharmacokinetic properties [2,3]. So far, five NNRTIs drugs—nevirapine, delavirdine, efavirenz, etravirine and rilpivirine (Figure 1)—have been approved for clinical use [4]. However, the high mutation rate of HIV-1 RT and the rapid emergence of drug resistance has limited the therapeutic efficacy of NNRTIs and this necessitates the continued discovery and development of drugs with novel mechanisms of action [5].

Sulfo-dihydro-alkylthio-benzyl-oxopyrimidines (S-DABOs) as representative NNRTIs [6,7], show their activity not only on HIV-1 wild type, but also on HIV-1 mutants [8]. By studying the crystal structures of different kinds of NNRTIs, it’s found that S-DABOs share the same binding mode as that of TNK-651. They interact with the binding site composed by Tyr181, Tyr188 and Trp229 residues, which is near the catalytic site of the RT. The N-3-H of S-DABOs forms a crucial hydrogen bond with Lys101 inside the hydrophobic pocket. In addition, the molecular may rotate to a butterfly-like conformation so that the crucial hydrophobic forces can be formed between the aryl moiety at C-2 and C-6 and the amino acid residues such as Tyr181, Tyr188, Phe227 [9,10,11,12,13,14].

Based on these promising results, some modifications were performed to achieve more active S-DABO derivatives. It was anticipated that an oxygen atom on the C-2 side chain would form an additional hydrogen bond, and extending the π-π conjugated system by introducing electron withdrawing groups at the *para* position of the phenyl ring could be beneficial for the activity [15]. Moreover, optimization in the C-6 phenylmethyl ring at the *meta* positions would lead to closer interactions with the non-nucleoside binding pocket (NNBP). On the basis of our previous results [16,17], the halogen atom on C-5 position could form an interaction with the carbonyl of Tyr181, which played an important role in improving anti-HIV-1 activity.

**Figure 2:**
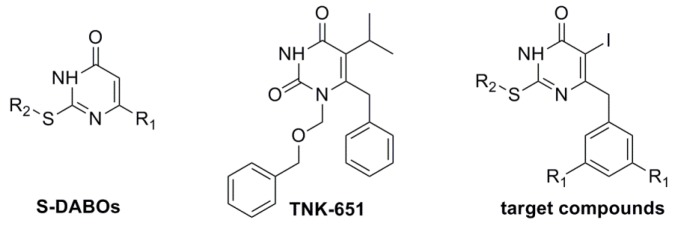
Structures of S-DABOs, TNK-651 and target compounds.

Herein, in order to verify the relationship between structure and activity of the target compounds, we synthesized a series of 2-arylalkylthio-5-iodine-6-substitutedbenzyl-pyrimidine-4(3*H*)-ones **8a**–**x** (Figure 2), and evaluated their inhibitory activity on HIV-1 RT and HIV-1SF33 in TZM-bl cell lines.

## 2. Results and Discussion

### 2.1. Chemistry

**Scheme 1:**
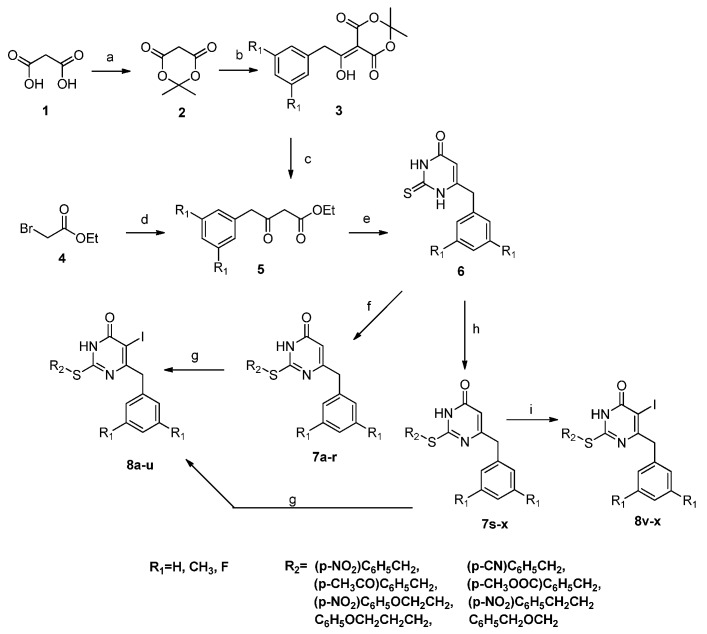
Synthesis of Target Compounds **8a**–**x**.

A general synthetic route to the target compounds is outlined in Scheme 1. The β-ketoesters 5 were prepared via phenylacetyl chloride and Meldrum’s acid without substituents on the phenyl ring [18,19]. Alternatively, for the compounds with dimethyl or difluoro *m*-substituents on the aromatic moiety, the corresponding compound 5 was generally obtained by reaction of the appropriate substituted benzyl cyanide with zinc and ethyl 2-bromoacetate using the method of Hannick and Kishi [20]. Condensation of 5 with thiourea in the presence of sodium ethoxide in boiling ethanol provided the 6-aryl thiouracils 6. The S-DABOs analogues **7a**–**x** were prepared by S-alkylation using the appropriate substituted alkyl halide and potassium carbonate in anhydrous DMF or sodium ethoxide in ethanol [21,22,23]. Subsequent treatment of **7a**–**u** with I_2_ and (NH_4_)_2_Ce(NO_3_)_6_ (CAN) in refluxing CH_3_CN afforded the corresponding target compounds **8a**–**u** [24]. Alternatively, another iodination method with NIS in DMF at 0 °C led to target compounds **8v**–**x** [25]. The structure assignments of these compounds were verified by NMR and mass spectral data.

*Reagents and conditions*: (a) acetone, acetic anhydride, con.H_2_SO_4_, rt; (b) PhCH_2_COCl, pyridine, CH_2_Cl_2_, ice bath; (c) EtOH, reflux; (d) *m*-difluoro/*m*-dimethyl benzyl cyanide, Zn/THF, reflux; (e) thiourea, EtONa/EtOH, reflux; (f) substituted alkyl halide, K_2_CO_3_, rt; (g) I_2_, CAN, reflux; (h) substituted alkyl halide, MeONa/MeOH, rt; (i) NIS, DMF, ice bath.

### 2.2. Biological Evaluation

All target compounds were evaluated as inhibitors of HIV-1 RT and some active compounds were evaluated for anti-HIV-1_SF33_ activity in the TZM-bl cell line with nevirapine and TNK-651 as reference compounds (Table 1 and Table 2) [26]. The biological results revealed that some compounds had potent activity and exhibited obvious structure-activity relationships.

**Table 1:** Inhibitory Activity of **7a**–**8x** against HIV-1 RT ^a^ 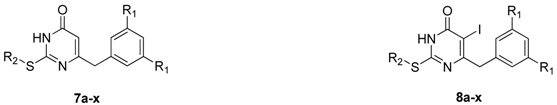

Compd.	R_1_	R_2_	IC_50_^b^ µM	Compd	R_1_	R_2_	IC_50_^b^ µM
**7a**	H	(p-NO_2_)C_6_H_5_CH_2_	>100	**8a**	H	(p-NO_2_)C_6_H_5_CH_2_	9.20
**7b**	CH_3_	(p-NO_2_)C_6_H_5_CH_2_	3.32	**8b**	CH_3_	(p-NO_2_)C_6_H_5_CH_2_	1.66
**7c**	F	(p-NO_2_)C_6_H_5_CH_2_	>100	**8c**	F	(p-NO_2_)C_6_H_5_CH_2_	5.50
**7d**	H	(p-CN)C_6_H_5_CH_2_	>100	**8d**	H	(p-CN)C_6_H_5_CH_2_	10.62
**7e**	CH_3_	(p-CN)C_6_H_5_CH_2_	29.63	**8e**	CH_3_	(p-CN)C_6_H_5_CH_2_	4.03
**7f**	F	(p-CN)C_6_H_5_CH_2_	15.77	**8f**	F	(p-CN)C_6_H_5_CH_2_	4.49
**7g**	H	(p-CH_3_CO)C_6_H_5_CH_2_	55.00	**8g**	H	(p-CH_3_CO)C_6_H_5_CH_2_	13.81
**7h**	CH_3_	(p-CH_3_CO)C_6_H_5_CH_2_	17.04	**8h**	CH_3_	(p-CH_3_CO)C_6_H_5_CH_2_	0.41
**7i**	F	(p-CH_3_CO)C_6_H_5_CH_2_	33.92	**8i**	F	(p-CH_3_CO)C_6_H_5_CH_2_	4.03
**7j**	H	(p-CH_3_OOC)C_6_H_5_CH_2_	>100	**8j**	H	(p-CH_3_OOC)C_6_H_5_CH_2_	3.64
**7k**	CH_3_	(p-CH_3_OOC)C_6_H_5_CH_2_	7.50	**8k**	CH_3_	(p-CH_3_OOC)C_6_H_5_CH_2_	1.11
**7l**	F	(p-CH_3_OOC)C_6_H_5_CH_2_	>100	**8l**	F	(p-CH_3_OOC)C_6_H_5_CH_2_	0.71
**7m**	H	(p-NO_2_)C_6_H_5_CH_2_CH_2_	67.24	**8m**	H	(p-NO_2_)C_6_H_5_CH_2_CH_2_	8.64
**7n**	CH_3_	(p-NO_2_)C_6_H_5_CH_2_CH_2_	1.40	**8n**	CH_3_	(p-NO_2_)C_6_H_5_CH_2_CH_2_	0.70
**7o**	F	(p-NO_2_)C_6_H_5_CH_2_CH_2_	18.14	**8o**	F	(p-NO_2_)C_6_H_5_CH_2_CH_2_	6.88
**7p**	H	(p-NO_2_)C_6_H_5_OCH_2_CH_2_	4.58	**8p**	H	(p-NO_2_)C_6_H_5_OCH_2_CH_2_	6.28
**7q**	CH_3_	(p-NO_2_)C_6_H_5_OCH_2_CH_2_	0.59	**8q**	CH_3_	(p-NO_2_)C_6_H_5_OCH_2_CH_2_	1.31
**7r**	F	(p-NO_2_)C_6_H_5_OCH_2_CH_2_	1.31	**8r**	F	(p-NO_2_)C_6_H_5_OCH_2_CH_2_	2.56
**7s**	H	C_6_H_5_OCH_2_CH_2_CH_2_	16.70	**8s**	H	C_6_H_5_OCH_2_CH_2_CH_2_	5.88
**7t**	CH_3_	C_6_H_5_OCH_2_CH_2_CH_2_	2.64	**8t**	CH_3_	C_6_H_5_OCH_2_CH_2_CH_2_	12.92
**7u**	F	C_6_H_5_OCH_2_CH_2_CH_2_	14.42	**8u**	F	C_6_H_5_OCH_2_CH_2_CH_2_	18.03
**7v**	H	C_6_H_5_CH_2_OCH_2_	12.88	**8v**	H	C_6_H_5_CH_2_OCH_2_	10.20
**7w**	CH_3_	C_6_H_5_CH_2_OCH_2_	1.82	**8w**	CH_3_	C_6_H_5_CH_2_OCH_2_	8.75

^a^ Nevirapine and TNK-651 were used as reference compounds; IC_50_ value for nevirapine and TNK-651 is 6.29 µM and 0.14 µM respectively; ^b^ Compound dose (µM) required to inhibit the HIV-1 RT activity by 50%; Data represent mean values of three separate experiments, variation among triplicate samples was less than 15%.

In general, most of the tested compounds displayed activity against HIV-1 RT, and demonstrated a lower IC_50_ than nevirapine within a range of 0.41 μM to 6.28 μM. As shown in Table 1, it was noteworthy that the activity of compounds **8a**–**o**, without an oxygen atom on the C-2 side chain, was much better than that of **7a**–**o**, especially **8h** (IC_50_ = 0.41 µM), **8l** (IC_50_ = 0.71 µM) and **8n** (IC_50_ = 0.70 µM), were about 10-fold more active compared to nevirapine (IC_50_ = 6.29 µM). Interestingly, the conclusion did not apply to **7p**–**x** which had oxygen atom on the C-2 side chain. On the contrary, **7p**–**x** revealed better activities than **8p**–**x**, such as **7q** > **8q**, **7r** > **8r**, **7t** > **8t** and **7w** > **8w**. Therefore, it was suggested that the oxygen atom might play a crucial role in the binding process with HIV-1 RT. In addition, substituting electron withdrawing groups at the *para* position of the phenyl ring at C-2 while increasing the length of side chain, would both be beneficial for the inhibitory activity, as exemplified by **7q** > **7n** > **7b**, **8p** > **8m** > **8a**, which were consistent with the previous work in our lab [16,17]. Meanwhile, taking **8n** > **8o** > **8m** and **8q** > **8r** > **8p** as examples, the activities were significantly improved by incorporating dimethyl and difluoro into the *meta* position of the C-6 aromatic moiety. According to the combined results, the target compounds **8h** (IC_50_ = 0.41 µM), **8l** (IC_50_ = 0.71 µM), **8n** (IC_50_ = 0.70 µM), **7q** (IC_50_ = 0.59 µM) had the best activities.

**Table 2:** Antiviral Activity of Compounds on HIV-1SF33 Infection in TZM-bl Cell line.

Compd.	MW	EC_50_ (μM)/SF33	Compd.	MW	EC_50 _ (μM)/SF33
**7n**	395	6.12	**8n**	521	3.84
**7q**	411	5.07	**8q**	537	9.44
**8h**	504	1.06	**8r**	545	4.10
**NVP **^a^	265	0.19			

^a^ Nevirapine was used as reference compound.

Subsequently, the anti-HIV-1 activity of some active compounds was determined using nevirapine as reference compound. As shown in Table 2, the target compounds were capable of inhibiting wild-type virus infection by HIV-1_SF33_ in TZM-bl cell line. Especially, **8h** showed the moderate activity. Meanwhile, the inhibitory activity of **7n** < **8n** and **7q** > **8q** was consistent with that of the RT assay. Moreover, the anti-HIV-1 cytotoxicity of these compounds was also determined and CC_50_values above 80 µM were observed.

### 2.3. Computational Modeling

**Figure 3:**
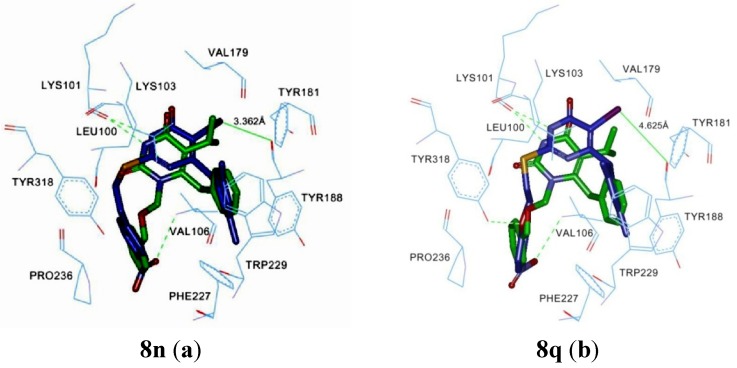
Binding mode of **8n** (**a**), **8q** (**b**) (blue) and TNK-651 (green) with HIV-1 RT. Hydrogen bonds are shown as a green dashed lines, and halogen bonds are shown as a green solid line.

Furthermore, in order to explore the structure-activity relationships, docking simulations were performed using the ChemScore program. Some target compounds were flexibly docked into the binding site of HIV-1 RT (PDB entry1RT2, in complex with TNK-651) (Figure 3). Default parameters were set according to the ChemScore manual, unless otherwise specified [27]. The results displayed that these target compounds could form a hydrogen bond between N-3-H and the amino acid residues Lys101 of RT, which was necessary for the activity. The series of compounds **7a**–**o** and **8a**–**o** had similar binding modes as the complex of TNK-651/1RT2. Compared to **7a**–**o**, **8a**–**o** a iodo substituent at the C-5 position could form an extra bond with the carbonyl of Tyr181, which had a significant impact on the activity by increasing the interaction. However, when an oxygen atom was introduced on the C-2 side chain, it could give different results. Another hydrogen bond was formed between the oxygen atom and the Tyr318 of RT. The extra hydrogen bond could potentially function as a pivot point to change the conformation of the compound when binding to the NNBP. Therefore, **7p**–**x** could interact more stronglywith RT than **7a**–**o**, which explained the observed increase of activity. Interestingly, as the conformation changed, the halogen bond at the C-5 position of **8p**–**x** disappeared unexpectedly. Herein, by forming the additional hydrogen bond, bearing an oxygen on the C-2 side chain could obviously affect the SAR of the traditional S-DABOs analogues. Moreover, to a certain extent, extending the C-2 side chain for a better location at the same region as N-1 substituent of TNK-651 was beneficial for the anti-HIV activity. Introducing substituents such as nitro, cyano, acetyl and ester, to the *para* position of the phenyl ring of the C-2 side chain, could significantly improve the inhibitory activity by forming another hydrogen bond with the Val106 residue. Meanwhile, because of the reduction in the electron density of the benzene ring with these electron withdrawing groups, a putative π-stacking interaction resulted with the electron rich benzene ring at the Tyr318 amino residue. In addition, due to the double or triple bonds in these groups, the well extended π-π conjugated system could increase the possibility of forming interactions with amino acid residues Phe227, Pro236 and so on. Considering the C-6 position, when the phenyl ring was characterized by *m*-dimethyl or *m*-difluoro substituents, favorable π-stacking and π-σ interactions would obviously be observed with the aromatic amino acid residues. Specifically, the dimethyl substituent could modulate the benzyl group into the most suitable conformation for the optimal affinity to the NNIBP by providing a special steric hindrance, where additional van der Waals contacts and π-σ interactions might be observed with the conserved Trp229 and Phe227 residues. Introducing a difluoro substituent at the benzene ring could reduce its electron density, which was beneficial for a better interaction by producing the π-stacking interaction.

## 3. Experimental

### 3.1. General Information

Melting points were measured on an X-4 apparatus and are reported without correction. NMR spectra were recorded on a Bruker AVANCE III-400 spectrometer with TMS as an internal standard and CDCl_3_ or DMSO-*d_6_* as the solvents. The chemical shifts were expressed in parts per million (δ ppm). Coupling constants (*J*) were measured in Hz. MS were taken by Bruker Apex IV FTMS with methanol as the solvent. FTIR spectra were recorded in the solid state as KBr dispersions using a Nicolet NEXUS 470 FTIR spectrometer. Silica gel H was used for the column chromatography and silica gel GF_254_ was used for TLC plates, which were used to monitor the reactions. CH_3_CN and acetone were dried with CaCl_2_, while other chemicals were purchased as reagent grade and used without further purification.

#### 3.1.1. General Procedure for the Syntheses of Compounds **7a**–**r**

6-Substitued benzyl-2-thioxouracil (compound **6**, 0.47 mmol) and K_2_CO_3_ (0.94 mmol) were dissolved in DMSO (2 mL) and stirred at room temperature, then the corresponding substituted alkyl bromide (0.47 mmol) was added to the solution followed by stirring for 12 h. H_2_O (7 mL) was added and then the pH of solution was adjusted to 3 with 2 N hydrochloric acid. After the addition of ethyl acetate (10 mL), the solution was stirred for another 15 min. Ethyl acetate (20 mL × 3) was used to extract the aqueous layer and the organic phases were combined, dried over MgSO_4_ and concentrated under reduced pressure with a rotary evaporator. The crude product was purified with silica gel H, and EtOAc–petroleum ether was used as the eluting solvent, to yield the target products **7a**–**r**.

*2-(4-Nitrobenzylthio)-6-benzyluracil* (**7a**). Yield: 62.61%. mp: 192–194 °C. ^1^H-NMR (DMSO-*d_6_*) δ: 3.77 (s, 2H, ArCH_2_), 4.40 (s, 2H, SCH_2_), 6.01 (s, 1H, 5-H), 7.19–7.29 (m, 5H, ArH), 7.47 (d, 2H, o-ArH, *J* = 8.4 Hz), 7.80 (d, 2H, m-ArH, *J* = 8.4 Hz). ^13^C-NMR (DMSO-*d_6_*) *δ*: 146.85, 138.33, 130.59, 129.69, 128.88, 126.94, 123.79 (C_arom_), 43.12 (SCH_2_), 32.83 (ArCH_2_). HRMS calcd. for C_18_H_16_N_3_O_3_S [M+H]^+^ 354.09069; found: 354.09007.

*2-(4-Nitrobenzylthio)-6-(3,5-dimethylbenzyl)uracil* (**7b**). Yield: 89.72%. mp: 218–220 °C. ^1^H-NMR (DMSO-*d_6_*) δ: 2.05 (s, 6H, Ar(CH_3_)_2_), 3.55 (s, 2H, (Me)_2_ArCH_2_), 4.27 (s, 2H, SCH_2_), 5.86 (s, 1H, 5-H), 6.70(s, 3H, (Me)_2_ArH), 7.35 (d, 2H, o-ArH, *J* = 8.4 Hz), 7.86(d, 2H, m-ArH, *J* = 8.4 Hz). ^13^C-NMR (DMSO-*d_6_*) *δ*: 162.28 (C-4), 159.86 (C-2), 158.00 (C-6), 149.79, 146.78, 138.14, 137.83, 130.58, 128.36, 127.39, 123.70 (C_arom_), 32.93 (SCH_2_), 30.21 (ArCH_2_), 21.28 (CH_3_). HRMS calcd. for C_20_H_20_N_3_O_3_S [M+H]^+^ 382.12199; found: 382.12142.

*2-(4-Nitrobenzylthio)-6-(3,5-difluorobenzyl)uracil* (**7c**). Yield: 73.05%. mp: 244–24 °C. ^1^H-NMR (DMSO-*d_6_*) δ: 3.79 (s, 2H, F_2_ArCH_2_), 4.42 (s, 2H, SCH_2_), 6.07 (s, 1H, 5-H), 6.93–7.06 (m, 3H, F_2_ArH), 7.52 (d, 2H, o-ArH, *J* = 8.4 Hz), 8.03 (d, 2H, m-ArH, *J* = 8.4 Hz). ^13^C-NMR (DMSO-*d_6_*) *δ*: 163.98 (C-4), 163.85 (C-F), 161.54 (C-2), 161.41 (C-6), 146.84, 146.68, 142.87, 130.39, 123.70, 112.86, 112.61 (C_arom_), 102.43 (C-5), 42.32 (SCH_2_), 33.07 (ArCH_2_). HRMS calcd. for C_18_H_14_F_2_N_3_O_3_S [M+H]^+^ 354.07184; found: 390.07125.

*2-(4-Cyanobenzylthio)-6-benzyluracil* (**7d**). Yield: 98.26%. mp: 190–193 °C. ^1^H-NMR (DMSO-*d_6_*) δ: 3.76 (s, 2H, ArCH_2_), 4.35 (s, 2H, SCH_2_), 6.00 (s, 1H, 5-H), 7.22–7.30 (m, 5H, ArH), 7.39 (d, 2H, o-ArH, *J* = 8.4 Hz), 7.61 (d, 2H, m-ArH, *J* = 8.4 Hz). ^13^C-NMR (DMSO-*d_6_*) *δ*: 144.62, 138.33, 132.59, 130.34, 129.67, 128.90, 126.98, 119.21 (C_arom_), 110.18 (CN), 43.18 (SCH_2_), 33.33 (ArCH_2_). HRMS calcd. for C_19_H_16_N_3_OS [M+H]^+^ 334.10086; found: 334.10041.

*2-(4-Cyanobenzylthio)-6-(3,5-dimethylbenzyl)uracil* (**7e**). Yield: 97.14%. mp: 193–196 °C. ^1^H-NMR (DMSO-*d_6_*) δ: 2.18 (s, 6H, Ar(CH_3_)_2_), 3.67 (s, 2H, (Me)_2_ArCH_2_), 4.37 (s, 2H, SCH_2_), 5.98 (s, 1H, 5-H), 6.83 (d, 3H, (Me)_2_ArH), 7.42 (d, 2H, o-ArH, *J* = 8 Hz), 7.60 (d, 2H, m-ArH, *J* = 8 Hz). ^13^C-NMR (DMSO-*d_6_*) *δ*: 144.65, 138.08, 137.813, 132.53, 130.34, 128.39, 127.39, 119.19 (C_arom_), 110.21 (CN), 43.08 (SCH_2_), 33.30 (ArCH_2_), 21.30 (CH_3_). HRMS calcd. for C_21_H_20_N_3_OS [M+H]^+^ 362.13216; found: 362.13153.

*2-(4-Cyanobenzylthio)-6-(3, 5-difluorobenzyl)uracil* (**7f**). Yield: 73.05%. mp: 205–208 °C. ^1^H-NMR (DMSO-*d_6_)* δ: 3.82 (s, 2H, F _2_ArCH_2_), 4.44 (s, 2H, SCH_2_), 6.70 (s, 1H, 5-H), 6.97–7.13 (m, 3H, F_2_ArH), 7.47 (d, 2H, o-ArH, *J* = 8.4 Hz), 7.66 (d, 2H, m-ArH, *J* = 8.4 Hz). ^13^C-NMR (DMSO-*d_6_*) *δ*: 164.00 (C-4), 163.86 (C–F), 161.55 (C-2), 161.42 (C-6), 144.52, 142.82, 132.52, 130.19, 119.17, 112.90, 112.66 (C_arom_), 110.26 (CN), 102.51 (C-5), 42.33 (SCH_2_), 33.32 (ArCH_2_). HRMS calcd. for C_19_H_14_ F_2_N_3_OS [M+H]^+^ 370.08202; found: 370.08147.

*2-(4-Acetylbenzylthio)-6-benzyluracil* (**7g**). Yield: 83.10%. mp: 204–205 °C. ^1^H-NMR (DMSO-*d_6_*) δ: 2.38 (s, 3H, ArCOCH_3_), 3.65 (s, 2H, ArCH_2_), 4.23 (s, 2H, SCH_2_), 5.87 (s, 1H, 5-H), 7.08–7.18 (m, 5H, ArH), 7.23 (d, 2H, o-ArH, *J* = 8.4 Hz), 7.62 (d, 2H, m-ArH, *J* = 8.4 Hz). ^13^C-NMR (DMSO-*d_6_*) *δ*: 197.84 (CO), 144.00, 138.36, 136.05, 129.68, 129.64, 128.88, 128.69, 126.94 (C_arom_), 43.22 (SCH_2_), 33.40 (ArCH_2_), 27.09 (CH_3_). HRMS calcd. for C_20_H_19_N_2_O_2_S [M+H]^+^ 351.11617; found: 351.11565.

*2-(4-Acetylbenzylthio)-6-(3,5-dimethylbenzyl)uracil* (**7h**). Yield: 85.18%. mp: 195–197 °C. ^1^H-NMR (DMSO-*d_6_*) δ: 2.23 (s, 6H, Ar(CH_3_)_2_), 2.54 (s, 3H, p-ArCOCH _3_), 3.73 (s, 2H, (Me)_2_ArCH_2_), 4.41 (s, 2H, SCH_2_), 6.01 (s, 1H, 5-H), 6.90 (s, 3H, (Me)_2_ArH), 7.41 (d, 2H, o-ArH, *J* = 7.2 Hz), 7.78 (d, 2H, m-ArH, *J* = 7.2 Hz). ^13^C-NMR (DMSO-*d_6_*) *δ*: 197.78 (CO), 150.27, 138.14, 137.82, 136.04, 129.64, 128.65, 128.38, 127.44 (C_arom_), 44.12 (SCH_2_), 43.15 (ArCH_2_), 27.06 (COCH_3_), 21.32 (CH_3_). HRMS calcd. for C_22_H_23_N_2_O_2_S [M+H]^+^ 379.14748; found: 379.14672.

*2-(4-Acetylbenzylthio)-6-(3,5-difluorobenzyl)uracil* (**7i**). Yield: 93.34%. mp: 251–254 °C. ^1^H-NMR (DMSO-*d_6_*) δ: 2.50 (s, 3H, ArCOCH_3_), 3.80 (s, 2H, F_2_ArCH_2_), 4.36 (s, 2H, SCH_2_), 6.05 (s, 1H, 5-H), 6.96–7.10 (m, 3H, F _2_ArH), 7.37 (d, 2H, o-ArH, *J* = 8 Hz), 7.76 (d, 2H, m-ArH, *J* = 8 Hz). ^13^C-NMR (DMSO-*d_6_*) *δ*: 197.75 (CO), 164.02 (C-4), 163.90 (C-F), 161.58 (C-2), 161.45 (C-6), 143.97, 142.88, 136.07, 129.50, 128.62, 112.95, 112.70 (C_arom_), 102.49 (C-5), 42.40 (SCH_2_), 33.39 (ArCH_2_), 27.04 (COCH_3_). HRMS calcd. for C_20_H_17_F_2_N_2_O_2_S [M+H]^+^ 387.09733; found: 387.09703.

*2-(4-Methoxycarbonylbenzylthio)-6-benzyluracil* (**7j**). Yield: 93.79%. mp: 214–217 °C. ^1^H-NMR (DMSO-*d_6_*) δ: 3.81 (s, 2H, ArCH_2_), 3.85 (s, 3H, ArCOOCH_3_), 4.40 (s, 2H, SCH_2_), 6.03 (s, 1H, 5-H), 7.25–7.34 (m, 5H, ArH), 7.39 (d, 2H, o-ArH, *J* = 8 Hz), 7.80 (d, 2H, m-ArH, *J* = 8 Hz). ^13^C-NMR (DMSO-*d_6_*) *δ*: 166.52 (CO), 144.22, 138.33, 131.95, 129.71, 129.12, 128.86, 126.94 (C_arom_), 52.27 (OCH_3_), 43.04 (SCH_2_), 33.23 (ArCH_2_). HRMS calcd. for C_20_H_19_N_2_O_3_S [M+H]^+^ 367.11109; found: 367.11079.

*2-(4-Methoxycarbonylbenzylthio)-6-(3,5-dimethylbenzyl)uracil* (**7k**). Yield: 90.91%. mp: 221–224 °C. ^1^H-NMR (DMSO-*d_6_*) δ: 2.06 (s, 6H, Ar(CH_3_)_2_), 3.55 (s, 2H, (Me)_2_ArCH_2_), 3.68 (s, 3H, p-ArCO_2_CH_3_), 4.24 (s, 2H, SCH_2_), 5.84 (s, 1H, 5-H), 6.71 (s, 3H, (Me)_2_ArH), 7.23 (d, 2H, o-ArH, *J* = 8 Hz), 7.63 (d, 2H, m-ArH, *J* = 8 Hz). ^13^C-NMR (DMSO-*d_6_*) *δ*: 166.41 (CO), 144.30, 137.81, 129.71, 129.59, 128.74, 128.39, 127.43 (C_arom_), 52.52 (OCH_3_), 43.13 (SCH_2_), 33.34 (ArCH_2_), 21.30 (CH_3_). HRMS calcd. for C_22_H_23_N_2_O_3_S [M+H]^+^ 395.14239; found: 395.14195.

*2-(4-Methoxycarbonylbenzylthio)-6-(3,5-difluorobenzyl)uracil* (**7l**). Yield: 70.71%. mp: 279–280 °C. ^1^H-NMR (DMSO-*d_6_*) δ: 3.79 (s, 2H, F_2_ArCH_2_), 3.80 (s, 3H, p-ArCO_2_CH_3_), 4.35 (s, 2H, SCH_2_), 6.03 (s, 1H, 5-H), 6.97–7.07 (m, 3H, F _2_ArH), 7.37 (d, 2H, o-ArH, *J* = 8 Hz), 7.77 (d, 2H, m-ArH, *J* = 8 Hz). ^13^C-NMR (DMSO-*d_6_*) *δ*: 166.40 (CO), 144.21, 142.93, 129.57, 128.80, 112.96, 112.71 (C_arom_), 52.54 (OCH_3_), 42.38 (SCH_2_), 33.38 (ArCH_2_). HRMS calcd. for C_20_H_17_F_2_N_2_O_3_S [M+H]^+^ 403.09225; found: 403.09219.

*2-(4-Nitrophenethylthio)-6-benzyluracil* (**7m**). Yield: 84.14%. mp: 200–204 °C. ^1^H-NMR (DMSO-*d_6_*) δ: 3.01 (t, 2H, p-NO_2_ArCH_2_, *J* = 7.2 Hz), 3.34 (t, 2H, J 6.8Hz, SCH_2_, *J* = 7.2 Hz), 3.80 (s, 2H, ArCH_2_), 5.98 (s, 1H, 5-H), 7.20–7.32 (m, 5H, ArH), 7.43 (d, 2H, o-ArH, *J* = 8 Hz), 8.13 (d, 2H, m-ArH, *J* = 8 Hz). ^13^C-NMR (DMSO-*d_6_*) *δ*: 148.57, 146.64, 138.41, 130.32, 129.75, 128.71, 127.02, 123.87 (C_arom_), 43.10 (SCH_2_), 35.11 (ArCH_2_), 30.64 (NO_2_ArCH_2_). HRMS calcd. for C_19_H_18_N_3_O_3_S [M+H]^+^ 368.10634; found: 368.10628.

2-(4-Nitrophenethylthio)-6-(3,5-dimethylbenzyl)uracil (**7n**). Yield: 50.20%. mp: 212–215 °C. ^1^H-NMR (DMSO-*d_6_*) δ: 2.02 (s, 6H, Ar(CH_3_)_2_), 2.88 (t, 2H, p-NO_2_ArCH_2_, *J* = 7.2 Hz), 3.19 (t, 2H, SCH_2_, *J* = 7.2 Hz), 3.55 (s, 2H, (Me)_2_ArCH_2_), 5.81 (s, 1H, 5-H), 6.70 (d, 3H, (Me)_2_ArH), 7.28 (d, 2H, o-ArH, *J* = 7.2 Hz), 7.98(d, 2H, m-ArH, *J* = 7.2 Hz). ^13^C-NMR (DMSO-*d_6_*) *δ*: 146.62, 137.73, 130.34, 128.32, 127.38, 123.85 (C_arom_), 46.63 (SCH_2_), 35.01 (ArCH_2_), 30.64 (NO_2_ArCH_2_), 21.25 (CH_3_). HRMS calcd. for C_21_H_22_N_3_O_3_S [M+H]^+^ 396.13764; found: 396.13726. IR (KBr): 3433, 3114, 2917, 2852, 1646, 1605, 1522, 1459, 1346, 837, 756, 729 cm^−1^.

*2-(4-Nitrophenethylthio)-6-(3,5-difluorobenzyl)uracil* (**7o**). Yield: 79.25%. mp: 220–222 °C. ^1^H-NMR (DMSO-*d_6_*) δ: 2.98 (t, 2H, p-NO_2_ArCH_2_, *J* = 7.6 Hz), 3.30 (t, 2H, SCH_2_, *J* = 7.6 Hz), 3.81 (s, 2H, F_2_ArCH_2_), 6.02 (s, 1H, 5-H), 7.00–7.04 (m, 3H, F _2_ArH), 7.41 (d, 2H, o-ArH, *J* = 8.4 Hz), 8.11 (d, 2H, m-ArH, *J* = 8.4 Hz). ^13^C-NMR (DMSO-*d_6_*) *δ*: 164.00 (C-4), 163.87 (C–F), 161.42 (C-6), 148.52, 146.64, 142.92, 130.28, 123.83, 112.96, 112.71 (C_arom_), 102.73 (C-5), 42.41 (SCH_2_), 35.00 (ArCH_2_), 30.62 (NO_2_ArCH_2_). HRMS calcd. for C_19_H_16_F_2_N_3_O_3_S [M+H]^+^ 404.08749; found: 404.08743.

*2-(2-(4-Nitrophenoxy)ethylthio)-6-benzyluracil* (**7p**). Yield: 63.46%. mp: 212–213 °C. ^1^H-NMR (DMSO-*d_6_*) δ: 3.49 (t, 2H, p-NO_2_ArCH_2_), 3.76 (s, 2H, ArCH_2_), 4.25 (t, 2H, SCH_2_), 5.99 (s, 1H, 5-H), 7.18 (d, 2H, o-ArH, *J* = 8 Hz), 7.16–7.29 (m, 5H, ArH), 8.18 (d, 2H, m-ArH, *J* = 8 Hz). ^13^C-NMR (DMSO-*d_6_*) *δ*: 163.80 (CO), 141.46, 138.20, 129.62, 128.79, 126.90, 126.28, 115.47 (C_arom_), 67.30 (OCH_2_), 42.87 (SCH_2_), 28.76 (ArCH_2_), 21.25 (CH_3_). HRMS calcd. for C_19_H_18_N_3_O_4_S [M+H]^+^ 384.10125; found: 384.10106.

2-(2-(4-Nitrophenoxy)ethylthio)-6-(3,5-dimethylbenzyl)uracil (**7q**). Yield: 82.12%. mp: 210–214 °C. ^1^H-NMR (DMSO-*d_6_*) δ: 2.16 (s, 6H, Ar(CH_3_)_2_), 3.46 (t, 2H, SCH_2_, *J* = 6.4 Hz), 3.65 (s, 2H, (Me)_2_ArCH_2_), 4.24 (t, 2H, OCH_2_, *J* = 6.4 Hz), 5.95 (s, 1H, 5-H), 6.80 (d, 3H, (Me)_2_ArH), 7.08 (d, 2H, o-ArH, *J* = 9.2 Hz), 8.15 (d, 2H, m-ArH, *J* = 9.2 Hz). ^13^C-NMR (DMSO-*d_6_*) *δ*: 163.78 (CO), 141.43, 138.00, 137.72, 128.32, 127.36, 126.27, 115.45 (C_arom_), 67.22 (OCH_2_), 43.02 (SCH_2_), 28.84 (ArCH_2_), 21.25 (CH_3_). HRMS calcd. for C_21_H_22_N_3_O_4_S [M+H]^+^ 412.13255; found: 412.13196. IR (KBr): 3433, 2920, 2851, 1648, 1595, 1546, 1513, 1465, 1345, 1266, 1027, 843, 751, 652 cm^−^^1^.

*2-(2-(4-Nitrophenoxy)ethylthio)-6-(3,5-difluorobenzyl)uracil* (**7r**). Yield: 82.76%. mp: 210–212 °C. ^1^H-NMR (DMSO-*d_6_*) δ: 3.48 (t, 2H, SCH_2_, *J* = 6.4 Hz), 3.81 (s, 2H, F_2_ArCH_2_), 4.24 (t, 2H, OCH_2_, *J* = 6.4 Hz), 6.07 (s, 1H, 5-H), 6.99–7.02 (m, 3H, F_2_ArH), 7.09 (d, 2H, o-ArH, *J* = 9.2 Hz), 8.18 (d, 2H, m-ArH, *J* = 9.2 Hz). ^13^C-NMR (DMSO-*d_6_*) *δ*: 163.94 (C-4), 163.78 (CO), 163.21 (C–F), 161.49 (C-2), 161.36 (C-6), 143.35, 142.76, 141.44, 126.27, 115.39, 112.92, 112.68 (C_arom_), 102.43 (C-5), 67.24 (OCH_2_), 43.29 (SCH_2_), 28.90 (ArCH_2_). HRMS calcd. for C_19_H_16_F_2_N_3_O_4_S [M+H]^+^ 420.08241; found: 420.08188.

#### 3.1.2. General Procedure for the Syntheses of Compounds **7s**–**x**

Sodium (6.43 mmol)) was dissolved in anhydrous methanol (20 mL), 6-substitued benzyl-2-thiouracil (compound **6**, 0.92 mmol) was added, followed by addition of the appropriate substituted alkyl halide (1.38 mmol). After completion of the reaction according to TLC analysis, the crude product were purified by silica gel column chromatography with EtOAc–petroleum ether as eluent to afford the white solid products **7s**–**x**.

*2-(Phenoxypropylthio)-6-benzyluracil* (**7s**). Yield: 79.17%. mp: 89–91 °C. ^1^H-NMR (CDCl_3_) δ: 2.11–2.17 (m, 2H, ArOCH_2_CH_2_), 3.36 (t, 2H, SCH_2_, *J* = 7.2 Hz), 3.79 (s, 2H, ArCH_2_), 4.02 (t, 2H, ArOCH_2_, *J* = 7.2 Hz), 5.99 (s, 1H, 5-H), 6.89–7.33 (m, 10H, ArH). ^13^C-NMR (DMSO-*d_6_*) *δ*: 158.85 (CO), 138.17, 129.95, 129.72, 128.78, 126.91, 121.03, 114.88 (C_arom_), 66.51 (OCH_2_), 43.16 (SCH_2_), 29.27 (ArCH_2_), 27.11 (CH_2_). HRMS calcd. for C_20_H_21_N_2_O_2_S [M+H]^+^ 353.13183; found: 353.13142.

*2-(Phenoxypropylthio)-6-(3,5-dimethylbenzyl)uracil* (**7t**). Yield: 64.57%. mp: 100–102 °C. ^1^H-NMR (CDCl_3_) δ: 2.12–2.18 (m, 2H, ArOCH_2_CH_2_), 2.17 (s, 6H, Ar(CH_3_)_2_), 3.35 (t, 2H, SCH_2_, *J* = 7.2 Hz), 3.69 (s, 2H, (Me)_2_ArCH_2_), 4.01 (t, 2H, ArOCH_2_, *J* = 7.2 Hz), 5.96 (s, 1H, 5-H), 6.84–6.96 (m, 5H, ArH), 7.29 (d, 3H, (Me)_2_ArH). ^13^C-NMR (DMSO-*d_6_*) *δ*: 158.97 (CO), 138.01, 137.74, 129.84, 128.32, 127.45, 121.02, 114.83 (C_arom_), 66.21 (OCH_2_), 43.17 (SCH_2_), 28.76 (ArCH_2_), 26.92 (CH_2_), 21.38 (CH_2_). HRMS calcd. for C_22_H_25_N_2_O_2_S [M+H]^+^ 381.16313; found: 381.16260.

*2-(Phenoxypropylthio)-6-(3,5-difluorobenzyl)uracil* (**7u**). Yield: 62.14%. mp: 100–101 °C. ^1^H-NMR (CDCl_3_) δ: 2.09–2.16 (m, 2H, ArOCH_2_CH_2_), 3.33 (t, 2H, SCH_2_, *J* = 7.2 Hz), 3.72 (s, 2H, F_2_ArCH_2_), 4.01 (t, 2H, ArOCH_2_, *J* = 7.2 Hz), 5.99 (s, 1H, 5-H), 6.67–7.30 (m, 8H, ArH). ^13^C-NMR (DMSO-*d_6_*) *δ*: 163.91 (C-F), 161.02 (C-6), 158.78 (CO), 143.00, 129.81, 121.02, 114.83, 113.02, 102.57 (C_arom_), 65.87 (OCH_2_), 42.40 (SCH_2_), 29.12 (ArCH_2_), 27.27 (CH_2_). HRMS calcd. for C_20_H_19_F_2_N_2_O_2_S [M+H]^+^ 389.11298; found: 389.11317.

2-(Benzyloxymethylthio)-6-benzyluracil (**7v**). Yield: 72.62%. mp: 118–120 °C. ^1^H-NMR (CDCl_3_) δ: 3.82 (s, 2H, ArCH_2_), 4.60 (s, 2H, ArCH_2_O), 5.43 (s, 2H, SCH_2_O), 5.99 (s, 1H, 5-H), 7.23–7.37 (m, 10H, ArH). ^13^C-NMR (DMSO-*d_6_*) *δ*: 176.47 (C-4), 161.01 (C-2), 156.20 (C-6), 136.03, 129.53, 129.09, 128.73, 128.26, 127.91, 127.48 (C_arom_), 104.14 (C-5), 70.93 (OCH_2_), 69.19 (SCH_2_), 27.56 (ArCH_2_). HRMS calcd. for C_19_H_19_N_2_O_2_S [M+H]^+^ 339.11617; found: 339.11591.

*2-(Benzyloxymethylthio)-6-(3,5-dimethylbenzyl)uracil* (**7w**). Yield: 65.13%. mp: 134–136 °C. ^1^H-NMR (CDCl_3_) δ: 2.17 (s, 6H, Ar(CH_3_)_2_), 3.73 (s, 2H, ArCH_2_), 4.58 (S, 2H, ArCH_2_O), 5.42 (s, 2H, SCH_2_O), 5.98 (s, 1H, 5-H), 6.83 (d, 3H, (Me)_2_ArH), 7.26–7.35 (m, 5H, ArH). ^13^C-NMR (DMSO-*d_6_*) *δ*: 156.20 (C-6), 138.07, 137.73, 128.69, 128.38, 128.16, 128.11, 127.42, 127.23 (C_arom_), 103.73 (C-5), 71.72 (OCH_2_), 70.93 (SCH_2_), 37.13 (ArCH_2_), 21.16 (CH_3_). HRMS calcd. for C_21_H_23_N_2_O_2_S [M+H]^+^ 367.14748; found: 367.14662.

*2-(Benzyloxymethylthio)-6-(3,5-difluorobenzyl)uracil* (**7x**). Yield: 57.77%. mp: 120–122 °C. ^1^H-NMR (CDCl_3_) δ: 3.78 (s, 2H, F_2_ArCH_2_), 4.62 (S, 2H, ArCH_2_O), 5.42 (s, 2H, SCH_2_O), 6.03 (s, 1H, 5-H), 6.68 (d, 3H, F _2_ArH), 7.28–7.37 (m, 5H, ArH). ^13^C-NMR (DMSO-*d_6_*) *δ*: 176.47 (C-4), 163.59 (C-F), 161.34 (C-2), 154.78 (C-6), 140.83, 137.83, 128.73, 127.56, 112.98, 112.73, 102.53 (C_arom_), 104.55 (C-5), 71.79 (OCH_2_), 70.63 (SCH_2_), 36.49 (ArCH_2_). HRMS calcd. for C_19_H_17_F_2_N_2_O_2_S [M+H]^+^ 375.09733; found: 375.09789.

#### 3.1.3. General Procedure for the Syntheses of Compounds **8a**–**u**

Compound **7a**–**u** (0.30 mmol) was dissolved in dry CH_3_CN (20 mL) and stirred at room temperature, then I_2_ (0.18 mmol) and Ce(NH_4_)_2_(NO_3_)_6_ (CAN, 0.18 mmol) were added to this solution followed by refluxing for 4 h. After cooling to room temperature, the reaction mixture was quenched with saturated aqueous NaHSO_3_ (15 mL). The CH_3_CN was evaporated under reduced pressure, and the residue was extracted with CH_2_Cl_2_ (20 mL × 3). The organic phases were combined, dried over MgSO_4_ and concentrated at reduced pressure with a rotary evaporator. The crude product was purified with silica gel H, and EtOAc–petroleum ether was used as the eluting solvent, to yield products **8a**–**u**.

*2-(4-Nitrobenzylthio)-5-iodine-6-benzyluracil* (**8a**). Yield: 75.74%. mp: 207–210 °C. ^1^H-NMR (DMSO-*d_6_*) δ: 4.11 (s, 2H, ArCH_2_), 4.41 (s, 2H, SCH_2_), 7.24–7.30 (m, 5H, ArH), 7.45 (d, 2H, o-ArH, *J* = 8.4 Hz), 8.01 (d, 2H, m-ArH, *J* = 8.4 Hz). ^13^C-NMR (DMSO-*d_6_*) *δ*: 146.87, 146.47, 137.70, 130.49, 129.36, 128.93, 127.03, 123.78 (C_arom_), 46.41 (SCH_2_), 32.02 (ArCH_2_). HRMS calcd. for C_18_H_15_IN_3_O_3_S [M+H]^+^ 479.98733; found: 479.98704.

*2-(4-Nitrobenzylthio)-5-iodine-6-(3,5-dimethylbenzyl)uracil* (**8b**). Yield: 79.90%. mp: 224–228 °C. ^1^H-NMR (DMSO-*d_6_*) δ: 2.16 (s, 6H, Ar(CH_3_)_2_), 4.01 (s, 2H, (Me)_2_ArCH_2_), 4.38 (s, 2H, SCH_2_), 6.816 (s, 3H, (Me)_2_ArH),7.41 (d, 2H, o-ArH, *J* = 8.4 Hz), 7.95 (d, 2H, m-ArH, *J* = 8.4 Hz). ^13^C-NMR (DMSO-*d_6_*) *δ*: 146.79, 146.53, 137.78, 137.50, 130.46, 128.46, 127.07, 123.66 (C_arom_), 46.56 (SCH_2_), 33.11 (ArCH_2_), 21.33 (CH_3_). HRMS calcd. for C_20_H_19_IN_3_O_3_S [M+H]^+^ 508.01863; found: 508.01826.

*2-(4-Nitrobenzylthio)-5-iodine-6-(3,5-difluorobenzyl)uracil* (**8c**). Yield: 69.81%. mp: 203–206 °C. ^1^H-NMR (DMSO-*d_6_*) δ: 4.10 (s, 2H, F_2_ArCH_2_), 4.40 (s, 2H, SCH_2_), 6.85–7.05 (m, 3H, F_2_ArH), 7.50 (d, 2H, o-ArH, *J* = 8.8 Hz), 8.04 (d, 2H, m-ArH, *J* = 8.8 Hz). ^13^C-NMR (DMSO-*d_6_*) *δ*: 163.85 (C-F), 146.83, 146.35, 142.00, 130.28, 123.68, 112.60, 112.35 (C_arom_), 102.51 (C-5), 47.78 (SCH_2_), 33.22 (ArCH_2_). HRMS calcd. for C_18_H_13_F_2_IN_3_O_3_S [M+H]^+^ 515.96849; found: 515.96792.

*2-(4-Cyanobenzylthio)-5-iodine-6-benzyluracil* (**8d**). Yield: 78.26%. mp: 223–226 °C. ^1^H-NMR (DMSO-*d_6_*) δ: 4.07 (s, 2H, ArCH_2_), 4.34 (s, 2H, SCH_2_), 7.21–7.29 (m, 5H, ArH), 7.35 (d, 2H, o-ArH, *J* = 7.2 Hz), 7.59 (d, 2H, m-ArH, *J* = 7.2 Hz), 13.05 (s, 1H, NH). ^13^C-NMR (DMSO-*d_6_*) *δ*: 144.26, 137.69, 132.55, 130.25, 129.37, 128.89, 127.08, 118.76 (C_arom_), 110.27 (CN), 46.79 (SCH_2_), 33.18 (ArCH_2_). HRMS calcd. for C_19_H_15_IN_3_OS [M+H]^+^ 459.99750; found: 459.99747.

*2-(4-Cyanobenzylthio)-5-iodine-6-(3,5-dimethylbenzyl)uracil* (**8e**). Yield: 65.47%. mp: 224–227 °C. ^1^H-NMR (DMSO-*d_6_*) δ: 2.17 (s, 6H, Ar(CH_3_)_2_), 3.99 (s, 2H, (Me)_2_ArCH_2_), 4.35 (s, 2H, SCH_2_), 6.83 (d, 3H, (Me)_2_ArH), 7.36 (d, 2H, o-ArH, *J* = 8 Hz), 7.57 (d, 2H, m-ArH, *J* = 8 Hz). ^13^C-NMR (DMSO-*d_6_*) *δ*: 144.29, 137.76, 137.46, 132.51, 130.23, 128.48, 127.08, 119.15 (C_arom_), 110.25 (CN), 46.53 (SCH_2_), 33.41 (ArCH_2_), 21.34 (CH_3_). HRMS calcd. for C_21_H_19_IN_3_OS [M+H]^+^ 428.02880; found: 488.02855.

*2-(4-Cyanobenzylthio)-5-iodine-6-(3,5-difluorobenzyl)uracil* (**8f**). Yield: 82.17%. mp: 216–219 °C. ^1^H-NMR (DMSO-*d_6_*) δ: 4.06 (s, 2H, F_2_ArCH_2_), 4.32 (s, 2H, SCH_2_), 6.83–7.08 (m, 3H, F_2_ArH), 7.38 (d, 2H, o-ArH, *J* = 8.4 Hz), 7.60 (d, 2H, m-ArH, *J* = 8.4 Hz). ^13^C-NMR (DMSO-*d_6_*) *δ*: 163.83 (C-F), 161.52 (C-2), 161.38 (C-6), 144.09, 142.01, 132.50, 130.06, 119.12, 112.64, 112.39 (C_arom_), 110.32 (CN), 102.56 (C-5), 46.12 (SCH_2_), 33.49 (ArCH_2_). HRMS calcd. for C_19_H_13_F_2_IN_3_OS [M+H]^+^ 495.97866; found: 495.97824.

*2-(4-Acetylbenzylthio)-5-iodine-6-benzyluracil* (**8g**). Yield: 86.51%. mp: 219–220 °C. ^1^H-NMR (DMSO-*d_6_*) δ: 2.52 (s, 3H, ArCOCH_3_), 4.10 (s, 2H, ArCH_2_), 4.35 (s, 2H, SCH_2_), 7.24–7.30 (m, 5H, ArH), 7.34 (d, 2H, o-ArH, *J* = 8 Hz), 7.76 (d, 2H, m-ArH, *J* = 8 Hz). ^13^C-NMR (DMSO-*d_6_*) *δ*: 197.68 (CO), 143.45, 137.64, 135.89, 129.57, 129.39, 128.88, 128.70, 127.05 (C_arom_), 46.45 (SCH_2_), 33.44 (ArCH_2_), 27.04 (CH_3_). HRMS calcd. for C_20_H_18_IN_2_O_2_S [M+H]^+^ 477.01282; found: 477.01248.

*2-(4-Acetylbenzylthio)-5-iodine-6-(3,5-Dimethylbenzyl)uracil* (**8h**). Yield: 79.97%. mp: 201–203 °C. ^1^H-NMR (DMSO-d_6_) δ: 2.20 (s, 6H, Ar(CH_3_)_2_), 2.53 (s, 3H, p-ArCOCH _3_), 4.04 (s, 2H, (Me)_2_ArCH_2_), 4.36 (s, 2H, SCH_2_), 6.89 (d, 3H, (Me)_2_ArH), 7.33 (d, 2H, o-ArH, *J* = 8 Hz), 7.75 (d, 2H, m-ArH, *J* = 8 Hz). ^13^C-NMR (DMSO-d_6_) δ: 197.72 (CO), 143.75, 137.78, 137.49, 136.06, 129.53, 128.63, 128.49, 127.13 (C_arom_), 46.59 (SCH_2_), 33.47 (ArCH_2_), 27.04 (COCH_3_), 21.36 (CH_3_). HRMS calcd. for C_22_H_22_IN_2_O_2_S [M+H]^+^ 505.04412; found: 505.04325. IR (KBr): 3434, 2920, 2851, 1728, 1675, 1605, 1449, 1354, 1266, 1120, 836, 776, 638, 615 cm^−1^.

*2-(4-Acetylbenzylthio)-5-iodine-6-(3,5-difluorobenzyl)uracil* (**8i**). Yield: 88.79%. mp: 205–206 °C. ^1^H-NMR (DMSO-*d_6_*) δ: 2.53 (s, 3H, ArCOCH_3_), 4.12 (s, 2H, F_2_ArCH_2_), 4.35 (s, 2H, SCH_2_), 6.93–7.11 (m, 3H, F _2_ArH), 7.35 (d, 2H, o-ArH, *J* = 8 Hz), 7.77 (d, 2H, m-ArH, *J* = 8 Hz). ^13^C-NMR (DMSO-*d_6_*) *δ*: 197.72 (CO), 164.00 (C-F), 161.54 (C-2), 161.41 (C-6), 143.59, 142.06, 136.05, 129.40, 128.60, 112.70, 112.45 (C_arom_), 102.57 (C-5), 46.17 (SCH_2_), 33.53 (ArCH_2_), 27.02 (COCH_3_). HRMS calcd. for C_20_H_16_F_2_IN_2_O_2_S [M+H]^+^ 512.99397; found: 512.99383.

*2-(4-Methoxycarbonylbenzylthio)-5-iodine-6-benzyluracil* (**8j**). Yield: 92.59%. mp: 214–216 °C. ^1^H-NMR (DMSO-*d_6_*) δ: 3.85 (s, 3H, p-ArCOOCH_3_), 4.12 (s, 2H, ArCH_2_), 4.38 (s, 2H, SCH_2_), 7.25–7.32 (m, 5H, ArH), 7.35 (d, 2H, o-ArH, *J* = 7.6 Hz), 7.78 (d, 2H, m-ArH, *J* = 7.6 Hz), 13.07 (s, 1H, NH). ^13^C-NMR (DMSO-*d_6_*) *δ*: 166.40 (CO), 143.82, 137.62, 129.64, 129.61, 129.40, 128.84, 127.03 (C_arom_), 52.69 (OCH_3_), 46.79 (SCH_2_), 33.53 (ArCH_2_). HRMS calcd. for C_20_H_18_IN_2_O_3_S [M+H]^+^ 493.00773; found: 493.00702.

*2-(4-Methoxycarbonylbenzylthio)-5-iodine-6-(3,5-dimethylbenzyl)uracil* (**8k**). Yield: 80.18%. mp: 208–211 °C. ^1^H-NMR (DMSO-*d_6_*) δ: 2.16 (s, 6H, Ar(CH_3_)_2_), 3.81 (s, 3H, p-ArCO_2_CH_3_), 4.01 (s, 2H, (Me)_2_ArCH_2_), 4.34 (s, 2H, SCH_2_), 6.84 (s, 3H, (Me)_2_ArH), 7.29 (d, 2H, o-ArH, *J* = 8 Hz), 7.72 (d, 2H, m-ArH, *J* = 8 Hz). ^13^C-NMR (DMSO-*d_6_*) *δ*: 166.37 (CO), 143.95, 137.77, 129.60, 129.58, 128.78, 128.49, 127.13 (C_arom_), 52.54 (OCH_3_), 46.60 (SCH_2_), 33.46 (ArCH_2_), 21.35 (CH_3_). HRMS calcd. for C_22_H_22_IN_2_O_3_S [M+H]^+^ 521.03903; found: 521.03770.

*2-(4-Methoxycarbonylbenzylthio)-5-iodine-6-(3,5-difluorobenzyl)uracil* (**8l**). Yield: 41.41%. mp: 210–213 °C. ^1^H-NMR (DMSO-*d_6_*) δ: 3.81(s, 3H, p-ArCO_2_CH_3_), 4.09 (s, 2H, F_2_ArCH_2_), 4.32 (s, 2H, SCH_2_), 6.88–7.08 (m, 3H, F_2_ArH), 7.32 (d, 2H, o-ArH, *J* = 8.4 Hz), 7.75 (d, 2H, m-ArH, *J* = 8.4 Hz). ^13^C-NMR (DMSO-*d_6_*) *δ*: 166.34 (CO), 163.86 (C-F), 161.54 (C-2), 161.41 (C-6), 143.80, 142.08, 129.53, 129.46, 128.81, 112.71, 112.46 (C_arom_), 52.53 (OCH_3_), 46.18 (SCH_2_), 33.49 (ArCH_2_). HRMS calcd. for C_20_H_16_F_2_IN_2_O_3_S [M+H]^+^ 528.98889; found: 528.98826.

*2-(4-Nitrophenethylthio)-5-iodine-6-benzyluracil* (**8m**). Yield: 75.83%. mp: 209–212 °C. ^1^H-NMR (DMSO-*d_6_*) δ: 2.93 (t, 2H, p-NO_2_ArCH_2_, *J* = 7.2 Hz), 3.28 (t, 2H, SCH_2_, *J* = 7.2 Hz), 4.09 (s, 2H, ArCH_2_), 7.18–7.30 (m, 5H, ArH), 7.35 (d, 2H, o-ArH, *J* = 8.4 Hz), 8.10 (d, 2H, m-ArH, *J* = 8.4 Hz), 12.99 (s, 1H, NH). ^13^C-NMR (DMSO-*d_6_*) *δ*: 148.36, 146.64, 137.87, 130.32, 129.28, 128.85, 127.02, 123.84 (C_arom_), 46.78 (SCH_2_), 34.84 (ArCH_2_), 30.80 (NO_2_ArCH_2_). HRMS calcd. for C_19_H_17_IN_3_O_3_S [M+H]^+^ 494.00298; found: 494.00270.

*2-(4-Nitrophenethylthio)-5-iodine-6-(3,5-dimethylbenzyl)uracil* (**8n**). Yield: 74.15%. mp: 220–221 °C. ^1^H-NMR (DMSO-*d_6_*) δ: 2.17 (s, 6H, Ar(CH_3_)_2_), 2.25 (t, 2H, p-NO_2_ArCH_2_, *J* = 7.6 Hz), 3.00 (t, 2H, SCH_2_, *J* = 7.6 Hz), 4.05 (s, 2H, (Me)_2_ArCH_2_), 6.88 (d, 3H, (Me)_2_ArH), 7.40 (d, 2H, o-ArH, *J* = 8.4 Hz), 8.12 (d, 2H, m-ArH, *J* = 8.4 Hz). ^13^C-NMR (DMSO-*d_6_*) *δ*: 148.47, 146.62, 137.72, 130.31, 128.43, 127.03, 126.49, 123.82 (C_arom_), 46.57 (SCH_2_), 34.81 (ArCH_2_), 30.86 (NO_2_ArCH_2_), 21.31 (CH_3_). HRMS calcd. for C_21_H_21_IN_3_O_3_S [M+H]^+^ 522.03428; found: 522.03387. IR (KBr): 3433, 2923, 2853, 1640, 1560, 1555, 1515, 1453, 1341, 1230, 1030, 995, 848, 711, 684, 596 cm^−1^.

*2-(4-Nitrophenethylthio)-5-iodine-6-(3,5-difluorobenzyl)uracil* (**8o**). Yield: 74.73%. mp: 220–221°C. ^1^H-NMR (DMSO-*d_6_*) δ: 2.94 (t, 2H, p-NO_2_ArCH_2_, *J* = 7.6 Hz), 3.27 (t, 2H, SCH_2_, *J* = 7.6 Hz), 4.14 (s, 2H, F_2_ArCH_2_), 6.98–7.08 (m, 3H, F _2_ArH), 7.39 (d, 2H, o-ArH, *J* = 8.8 Hz), 8.11 (d, 2H, m-ArH, *J* = 8.8 Hz). ^13^C-NMR (DMSO-*d_6_*) *δ*: 163.99 (C-4), 163.86 (C-F), 161.55 (C-6), 148.33, 146.63, 130.26, 123.79, 112.71, 112.46 (C_arom_), 102.53 (C-5), 46.20 (SCH_2_), 34.71 (ArCH_2_), 30.79 (NO_2_ArCH_2_). HRMS calcd. for C_19_H_15_F_2_IN_3_O_3_S [M+H]^+^ 529.98414; found: 529.98365.

*2-(2-(4-Nitrophenoxy)ethylthio))-5-iodine-6-benzyluracil* (**8p**). Yield: 63.64%. mp: 204–205 °C. ^1^H-NMR (DMSO-*d_6_*) δ: 3.44 (t, 2H, p-NO_2_ArOCH_2_, *J* = 6.4 Hz), 4.06 (s, 2H, ArCH_2_), 4.16 (t, 2H, SCH_2_, *J* = 6.4 Hz), 7.05 (d, 2H, o-ArH, *J* = 9.2 Hz), 7.16–7.29 (m, 5H, ArH), 8.16 (d, 2H, m-ArH, *J* = 9.2 Hz). ^13^C-NMR (DMSO-*d_6_*) *δ*: 163.91 (CO), 141.46, 137.86, 129.32, 128.31, 126.97, 126.14, 115.49 (C_arom_), 67.34 (OCH_2_), 46.80 (SCH_2_), 29.10 (ArCH_2_). HRMS calcd. for C_19_H_17_IN_3_O_4_S [M+H]^+^ 509.99791; found: 509.99834.

*2-(2-(4-Nitrophenoxy)ethylthio))-5-iodine-6-(3,5-dimethylbenzyl)uracil* (**8q**). Yield: 60.48%. mp: 215–218 °C. ^1^H-NMR (DMSO-*d_6_*) δ: 2.16 (s, 6H, Ar(CH_3_)_2_), 3.42 (t, 2H, SCH_2_, *J* = 6.8 Hz), 3.96 (s, 2H, (Me)_2_ArCH_2_), 4.18 (t, 2H, OCH_2_, *J* = 6.8 Hz), 6.81 (d, 3H, (Me)_2_ArH), 7.09 (d, 2H, o-ArH, *J* = 9.2 Hz), 8.15 (d, 2H, m-ArH, *J* = 9.2 Hz). ^13^C-NMR (DMSO-*d_6_*) *δ*: 163.72 (CO), 141.44, 137.68, 137.51, 128.41, 127.06, 126.23 (C_arom_), 115.39 (C-5), 67.02 (OCH_2_), 46.50 (SCH_2_), 29.18 (ArCH_2_), 21.31 (CH_3_). HRMS calcd. for C_21_H_21_IN_3_O_4_S [M+H]^+ ^538.02920; found: 538.02847. IR (KBr): 3433, 2920, 2851, 1633, 1596, 1554, 1513, 1464, 1347, 1258, 1231, 1030, 840, 750, 651, 596 cm^−1^.

*2-(2-(4-Nitrophenoxy)ethylthio)-5-Iodine-6-(3,5-difluorobenzyl)uracil* (**8r**). Yield: 75.52%. mp: 224–226 °C. ^1^H-NMR (DMSO-*d_6_*) δ: 3.41 (t, 2H, SCH_2_, *J* = 6.4 Hz), 4.09 (s, 2H, F_2_ArCH_2_), 4.15 (t, 2H, OCH_2_, *J* = 6.4 Hz), 6.98 (d, 3H, F_2_ArH), 7.05 (d, 2H, o-ArH, *J* = 9.2 Hz), 8.16 (d, 2H, m-ArH, *J* = 9.2 Hz). ^13^C-NMR (MHz, DMSO-*d_6_*) *δ*: 163.92 (CO), 163.73 (C-F), 161.48 (C-2), 161.34 (C-6), 141.43, 126.23, 115.36, 112.74, 112.49 (C_arom_), 102.49 (C-5), 67.03 (OCH_2_), 46.18 (SCH_2_), 29.17 (ArCH_2_). HRMS calcd. for C_19_H_14_F_2_IN_3_NaO_4_S [M+Na]^+^ 567.96100; found: 567.96056. IR (KBr): 3435, 2924, 2853, 1644, 1592, 1557, 1516, 1461, 1343, 1261, 1241, 1116, 1033, 851, 751, 656, 603 cm^−1^.

*2-(Phenoxypropylthio)-5-iodine-6-benzyluracil* (**8s**). Yield: 68.26%. mp: 135–137 °C. ^1^H-NMR (CDCl_3_) δ: 1.95–2.00 (m, 2H, ArOCH_2_CH_2_), 3.19–3.20 (m, 2H, SCH_2_), 3.82–3.90 (m, 2H, ArOCH_2_), 4.10 (s, 2H, ArCH_2_), 6.58–7.51 (m, 10H, ArH). ^13^C-NMR (DMSO-*d_6_*) *δ*: 158.85 (CO), 138.43, 129.95, 129.44, 128.90, 127.01, 121.04, 117.70 (C_arom_), 114.79 (C-5), 66.51 (OCH_2_), 46.73 (SCH_2_), 29.07 (ArCH_2_), 27.39 (CH_2_). HRMS calcd. for C_20_H_20_IN_2_O_2_S [M+H]^+^ 479.02847; found: 479.02956.

*2-(Phenoxypropylthio)-5-iodine-6-(3,5-dimethylbenzyl)uracil* (**8t**). Yield: 60.11%. mp: 165–167 °C. ^1^H-NMR (CDCl_3_) δ: 2.04–2.10 (m, 2H, ArOCH_2_CH_2_), 2.27 (s, 6H, Ar(CH_3_)_2_), 3.27 (t, 2H, SCH_2_, *J* = 7.2 Hz), 3.93 (t, 2H, ArOCH_2_, *J* = 7.2 Hz), 4.08 (s, 2H, (Me)_2_ArCH_2_), 6.86–7.04 (m, 5H, ArH), 7.27 (q, 3H, (Me)_2_ArH). ^13^C-NMR (DMSO-*d_6_*) *δ*: 158.80 (CO), 138.37, 137.63, 129.80, 128.28, 127.09, 120.98, 117.65 (C_arom_), 114.35 (C-5), 66.21 (OCH_2_), 46.04 (SCH_2_), 29.13 (ArCH_2_), 27.26 (CH_2_), 21.38 (CH_2_). HRMS calcd. for C_22_H_24_IN_2_O_2_S [M+H]^+^ 507.05977; found: 507.06077.

*2-(Phenoxypropylthio)-5-iodine-6-(3,5-difluorobenzyl)uracil* (**8u**). Yield: 62.40%. mp: 156–158 °C. ^1^H-NMR (CDCl_3_) δ: 2.05–2.11 (m, 2H, ArOCH_2_CH_2_), 3.26 (t, 2H, SCH_2_, *J* = 7.2 Hz), 3.99 (t, 2H, ArOCH_2_, *J* = 7.2 Hz), 4.10 (s, 2H, F_2_ArCH_2_), 6.66–6.97 (m, 5H, ArH), 7.28 (q, 3H, F_2_ArH). ^13^C-NMR (DMSO-*d_6_*) *δ*: 161.62 (C-F), 160.28 (C-6), 158.52 (CO), 142.26, 129.56, 120.79, 112.32, 112.03, 102.25 (C_arom_), 114.73 (C-5), 65.47 (OCH_2_), 46.36 (SCH_2_), 29.11 (ArCH_2_), 27.69 (CH_2_). HRMS calcd. for C_20_H_18_F_2_IN_2_O_2_S [M+H]^+^ 515.00962; found: 515.01071.

#### 3.1.4. General Procedure for the Syntheses of Compounds **8v**–**x**

Compound **7v**–**u** (0.10 mmol) and NIS (0.12 mmol) were dissolved in dry DMF (15 mL) after cooling to 0 °C of ice bath, then stirred to room temperature for another 10 h, the reaction completed according to TLC analysis. The mixture was added to EtOAc (50 mL) and washed by saturated brine (50 mL × 3). The organic phase was dried over MgSO_4_ and concentrated at reduced pressure with a rotary evaporator. The crude product was purified with silica gel H, and EtOAc–petroleum ether was used as the eluting solvent, to yield products **8v**–**x**.

*2-(Benzyloxymethylthio)-5-iodine-6-benzyluracil* (**8v**). Yield: 91.25%. mp: 133–135 °C. ^1^H-NMR (CDCl_3_) δ: 4.14 (s, 2H, ArCH_2_), 4.47 (s, 2H, ArCH_2_O), 5.35 (s, 2H, SCH_2_O), 7.15–7.33 (m, 8H, ArH). ^13^C-NMR (DMSO-*d_6_*) *δ*: 175.23 (C-4), 160.50 (C-2), 156.39 (C-6), 138.60, 129.12, 129.06, 128.80, 128.45, 127.49, 127.43 (C_arom_), 104.14 (C-5), 71.53 (OCH_2_), 69.84 (SCH_2_), 29.43 (ArCH_2_). HRMS calcd. for C_19_H_18_IN_2_O_2_S [M+H]^+^ 465.01282; found: 465.01307.

*2-(Benzyloxymethylthio)-5-iodine-6-(3,5-dimethylbenzyl)uracil* (**8w**). Yield: 78.90%. mp: 200–202 °C. ^1^H-NMR (CDCl_3_) δ: 2.20 (s, 6H, Ar(CH_3_)_2_), 4.07 (s, 2H, ArCH_2_), 4.47 (s, 2H, ArCH_2_O), 5.34 (s, 2H, SCH_2_O), 6.80–6.95 (m, 5H, ArH), 7.26–7.33 (m, 3H, (Me)_2_ArH). ^13^C-NMR (DMSO-*d_6_*) *δ*: 156.39 (C-6), 138.97, 137.49, 129.36, 129.02, 128.58, 128.06, 127.52, 126.96 (C_arom_), 103.73 (C-5), 71.72 (OCH_2_), 70.93 (SCH_2_), 37.13 (ArCH_2_), 21.16 (CH_3_). HRMS calcd. for C_21_H_22_IN_2_O_2_S [M+H]^+^ 493.04412; found: 493.04508.

*2-(Benzyloxymethylthio)-5-iodine-6-(3,5-difluorobenzyl)uracil* (**8x**). Yield: 78.93%. mp: 196–198 °C. ^1^H-NMR (CDCl_3_) δ: 4.12 (s, 2H, F_2_ArCH_2_), 4.59 (s, 2H, ArCH_2_O), 5.35 (s, 2H, SCH_2_O), 6.63–6.90 (m, 3H, F _2_ArH), 7.26–7.36 (m, 5H, ArH). ^13^C-NMR (DMSO-*d_6_*) *δ*: 176.62 (C-4), 163.46 (C-F), 161.48 (C-2), 154.41 (C-6), 141.21, 138.27, 128.83, 127.77, 112.99, 111.94, 102.59 (C_arom_), 105.25 (C-5), 72.22 (OCH_2_), 70.92 (SCH_2_), 35.53 (ArCH_2_). HRMS calcd. for C_19_H_16_F_2_IN_2_O_2_S [M+H]^+^ 500.99397; found: 500.99365.

### 3.2. Biological Assays

#### 3.2.1. Assay for Measuring the Inhibitory Activity of Compounds against HIV-1 RT

Oligo(dT) (TaKaRa Co., Otsu, Japan) was immobilized via its 5'-terminal phosphate to Covalink-NH microtiter plates (NUNC Co., Roskilde, Denmark). The biotin-dUTP was incorporated by reverse transcriptase (Sigma, St. Louis, MO, USA). Briefly, a serial concentration of inhibitor was added to the mixture, which contained 50 mmol/L Tris-HCl (pH 8.3), 3 mmol/L MgCl_2_, 75 mmol/L KCl, 5 mmol/L DTT, 0.13 mg/mL BSA, 10 μg/mL poly (A), 0.75 μM biotin-11-Dutp, 1.5 μM dTTP and HIV-1 RT. After incubation at 37 °C for 1h, washing buffer, which contained 50 mmol/L Tris-HCl (pH 7.5), 0.15 mol/L NaCl, 0.05 mmol/L MgCl_2_ and 0.02% Tween20, was used to wash the plate. Then each well of the plate was added 100 mL 1% BSA and incubated at room temperature for another 30 min. The plate was washed with the same buffer for 3 times. Before further incubation at 37 °C for 1 h, 50 μL of alkaline phosphatase streptavidin (SA-ALP) solution (100 ng/mL) was added to each well and washed again as above. Finally, 50 μL of *p*-nitrophenyl phosphate, disodium (PNPP, 1 mg/mL, pH 9.5) was added and incubated at 37 °C for 30 min. 0.5 mol/L NaOH was added to stop the reaction and a colorimetric streptavidin-alkaline phosphatase reporter system was used to detect and quantify the result.

#### 3.2.2. Assay for Measuring the Inhibitory Activity of Compounds on HIV-1_SF33_ Infectious

TZM-bl cells, HIV-1_SF33_ were obtained from the NIH AIDS Research and Reference Reagent Program (Germantown, MD, USA). The inhibitory activity of the compounds on infection by a laboratory-adapted HIV-1 strain SF33 was tested in TZM-bl cells. Briefly, TZM-bl cells (4 × 10^4^/well) were infected by addition of 200 TCID_50_ of HIV-1, followed by incubation for 2 h at 37 °C before addition of compounds at serial dilutions. After further incubation at 37 °C for 7 days, p24 was measured using a commercial enzyme-linked immunosorbent assay (ELISA) kit (Vironostika HIV-1 Microelisa system; BioMérieux; Marcy l’Etoile, France).The concentration of a compound for inhibiting 50% viral replication (EC_50_) was determined by nonlinear regression using GraphPad Prism 5.01.

### 3.3. Molecular Modeling

The docking studies were conducted by using software GOLD3.0.1 and protein crystal structures of wildtype RT/1 complex (PDB ID: 1RT2). Following the default settings of Chemscore in the software tool, top scored docked poses were visually inspected for each ligand in the DS 2.5 based on relevant molecules. The radius of the binding site sphere was defined by the original ligand as 8.9 Å, thus ensuring that the docking method was reliable.

## 4. Conclusions

In summary, a series of novel 2-arylalkylthio-5-iodine-6-substitutedbenzyl-pyrimidine-4(3*H*)-ones (S-DABOs) were synthesized as inhibitors of HIV-1 RT. We evaluated their anti-HIV activity on the HIV-1 RT and HIV-1_SF33_ in TZM-bl cell lines. Biological results revealed that the target compounds displayed good activity. It was noteworthy that compounds bearing an oxygen atom on the C-2 side chain demonstrated a new SAR, and the differences in biological results seen between **7a**–**x** and **8a**–**x** were discussed. From this study, useful information was obtained for designing HIV-1 inhibitors with great efficiency and low toxicity.
